# Concentration and Potential Human Health Hazards of Heavy Metals in Periwinkle (*Tympanotonus fuscatus*) Purchased from Major Markets in Calabar, Nigeria

**DOI:** 10.5696/2156-9614-10.28.201206

**Published:** 2020-12-02

**Authors:** Udiba Ugumanim Udiba, Udeme Uyom Udofia, Ekom R. Akpan

**Affiliations:** 1 Department of Zoology and Environmental Biology, University of Calabar, Calabar, Nigeria; 2z Institute of Oceanography, University of Calabar, Calabar, Nigeria

**Keywords:** estuarine filter, sink, lead, cadmium, chromium, nickel, periwinkle, health hazards

## Abstract

**Background.:**

As water flows through habitats associated with estuaries, such as mud flats, salt marshes, sea grass and mangrove forests, pollutants such as heavy metals are filtered. The fine sediment dominant in intertidal and subtidal estuarine systems is an important sink for these contaminants. Periwinkle, which inhabit estuarine ecosystems, are known to bioaccumulate large quantities of contaminants.

**Objectives.:**

In view of the widespread consumption of periwinkle in the Niger Delta, Nigeria, this study was designed to assess the concentration and potential human health hazards of heavy metals due to the consumption of this rich, inexpensive and readily available source of protein in Calabar, Nigeria.

**Methods.:**

Lead (Pb), cadmium (Cd), chromium (Cr) and nickel (Ni) content of edible tissues of periwinkles obtained from major markets in Calabar were determined using Shimadzu atomic absorption spectrophotometer (Model AA-6800, Japan) after wet digestion.

**Results.:**

The ranges of concentration (mg/kg dry weight) were Pb (0.011–0.056), Cd (0.008–0.032), Cr (0.014–0.157) and Ni (0.053–0.261) for Watt Market and Pb (0.009–0.052), Cd (0.011–0.032), Cr (0.012–0.052) and Ni (0.012–0.322) for Mariam Market. Concentrations of all the metals were below Food and Agricultural Organization (FAO), FAO/World Health Organization (WHO) and Commission of European Communities maximum permissible limits. The estimated daily intake (EDI) of Pb and Cd were slightly higher compared to the recommended daily intake for the metals. The EDI of all metals under study were lower than the upper tolerable daily intake. The target hazard quotients (THQ) computed to estimate the human health risk posed by each metal were above the safe limits of unity, except for Cr. The hazard index (HI) for a typical adult of 60.7 kg body weight was found to be 9.7 for Watt Market and the relative contributions to the aggregated risk were 24.66%, 54.51%, 0.0001% and 20.70% for Pb, Cd, Cr and Ni, respectively. The HI for Marian Market was 10.7 and the relative contributions to the aggregated risk were 22.31%, 57.55%, 0.06% and 20.09% for Pb, Cd, Cr and Ni, respectively.

**Conclusions.:**

Consumption of periwinkles purchased from major markets in Calabar poses toxicological risk with respect to Pb, Cd and Ni poisoning.

**Competing interests.:**

The authors declare no competing financial interests.

## Introduction

Fish and other seafood are an important source of human protein. In addition to the high protein content, the high omega-3 fatty acids, vitamins, essential mineral and the low saturated fat contents are known to contribute to good health. The American Heart Association recommends individuals eat fish at least twice a week in order to meet the recommended daily intake of omega-3 fatty acid.^1^ Fish have been recognized as good accumulators of organic and inorganic contaminants in the aquatic environment. Among different aquatic organisms, shellfish *(eg.* periwinkle (*Tympanotonus fuscatus)* and clam (*Egeria radiata))* accumulate large quantities of contaminants from their habitat due to their feeding habits and the nature of their habitats. They are found in muddy fresh water, the inter-tidal zone of brackish water, creeks and estuaries.^2^ Habitats associated with estuaries, such as salt marshes, mudflats, sea grass and mangrove forests act like enormous filters. As water flows through these habitats, pollutants such as herbicides, pesticides, heavy metals and plastics are filtered.^3^ The fine sediment dominant in intertidal and subtidal estuarine systems is an important short-term and long-term sink for these contaminants and therefore estuaries are one of the most polluted ecosystems.^3^

Heavy metal pollution of aquatic ecosystems is an environmental problem of global concern, partly because they are ubiquitous in nature and particularly due to their potential toxicity, non-biodegradability and persistence. Aquatic organisms are sensitive to heavy metals in water, sediments and food.^4^ Some heavy metals may bioaccumulate in certain food chains. Invertebrates such as shellfish, crabs and shrimps have been reported to bioaccumulate more heavy metals than fin fish because of differences in their evolutionary phylum-specific coping strategies.^5^ The two main ways through which heavy metals gain entry into the food chain are direct ingestion of water/food through the digestive track and non-dietary routes across the semi permeable membranes of the organism's tissues.^5^ Humans are at risk due to bioaccumulation and biomagnification of these toxic substances along the food chain. The rates of bioaccumulation and biomagnification of metals in aquatic organisms differ from one organism to another and depend on the type of metal concerned, chemical form of the metal and concentration in water/sediment.^4^ It follows that heavy metal concentrations measured in these organisms are a direct reflection of the concentrations in the medium from which they are sourced.^6^ The science of inferring the ecological condition of a given ecosystem by examining the organism inhabiting it (biomonitoring) is fast gaining attention as an indirect way of assessing human exposure to extraneous substances.^4^ At certain concentrations referred to as allowable limits, essential metals play vital roles in the human body as some of them form an integral part of enzymes that work for the proper functioning of the human system. Above allowable limits, even the most essential metal is capable of causing significant health challenges. Metals such as lead (Pb), cadmium (Cd) and mercury have no known significant biological role in the human body. They elicit extreme toxicity even at low concentrations and have been regarded as a serious threat to life. There is no limit below which these metals are said to be safe.^7^ Determining the health risk of seafood obtained from the Niger Delta estuaries is therefore of utmost importance to public health.

The periwinkle species *Tympanotonus fuscatus* is a deposit feeder, feeding on mud and digesting the detritus and other organic matter in highly productive and in most cases extremely polluted estuarine ecosystems. The organism is very sensitive to pollutants and has been previously used as a bio-indicator of aquatic pollution.^2^ *Tympanotonus fuscatus* is one of the most widely distributed species of periwinkles in the estuarine mud flats of southern Nigeria. The species is a delicacy in most riverine communities as they provide a relatively cheap source of animal protein especially for low-income earners and are often recommended for pregnant women and those who are protein deficient. The shells are used as a source of calcium in animal feeds and for construction purposes.

They are also painted and used as ornaments for decorations, forming an important industry in the Niger Delta region of Nigeria.^8^ The Niger Delta is continuously exposed to environmental pollution arising from oil exploration, exploitation, and its associated processing activities. Oil spills routinely occur in the oil rich region as part of the oil and gas exploration and exploitation process. An estimated 3.1 million barrels of crude oil enriched in manganese (Mn), iron (Fe), copper (Cu), zinc (Zn), Pb, nickel (Ni), cobalt, Cd and chromium (Cr) have been spilled between 1976 and 2014 in the region.^9^ Aquatic systems are the ultimate repositories of these contaminants given that over 90% of oil in Nigeria is drilled offshore. Elevated levels of Pb, Cd, Cr and Ni have been previously reported in the water column in the area.^9^ Tidal regimes have caused extensive distribution of pollutants across the length and breadth of the network of adjourning rivers, creeks and estuaries. In view of the wide consumption of periwinkle, this study was designed to assess the heavy metals content of this rich, cheap and readily available source of protein sold in the two major markets (Watt Market and Marian Market) in Calabar metropolis. Contaminant levels in food items can exceed the allowable limits set by regulatory authorities but may not present a significant risk to human health, probably due to the antagonistic effects of other contaminants. At other times, contaminant levels lower than allowable limits present significant deleterious effects due to synergy between different contaminants. Therefore, estimated dietary intake (EDI), target hazard quotient (THQ) and hazard index (HI) were used to evaluate potential health implications for consumers in order to safeguard public health.

**Abbreviations**
*ANOVA*: Analysis of variance
*bw*: Body weight
*EDI*: Estimated dietary intake
*FAO*: Food and Agricultural Organization
*HI*: Hazard index
*THQ*: Target hazard quotient
*USEPA*: United States Environmental Protection Agency
*WHO*: World Health Organization

**Figure 1:**
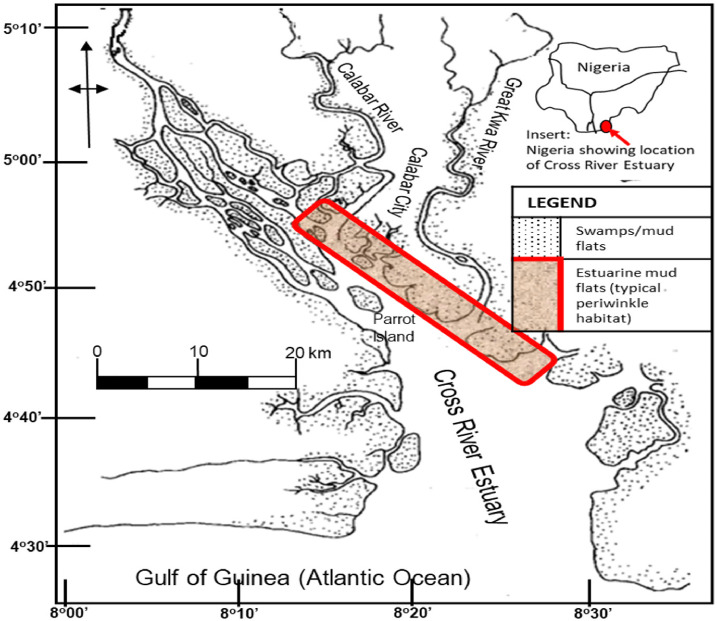
Map of study area showing typical periwinkle habitat

## Methods

Calabar Metropolis is the capital city of Cross River state (one of nine states in the oil rich Niger Delta region) of Nigeria with a population of 579,000 as of the year 2020.^10^ The city, although urbanized, does not have adequate waste management facilities.^11^ Municipal wastes and effluents are largely disposed of in open dumps and drains, most of which ends up in the adjoining rivers and swamps during the characteristic torrential rains. The city is drained by the Calabar River to the west and the Great Kwa River to the east, both of which discharge into the Cross River Estuary, which subsequently flows into the Atlantic Ocean at the Gulf of Guinea. Both rivers maintain a network of tributaries and creeks. The Cross River Estuary and its systems support a rich mangrove swamp ecosystem with extensive mud flats and swamps, rich in shell fishes, including periwinkles. *Tympanotonus fuscatus* is one of the commercially important periwinkle species harvested from this mangrove swamp and sold widely within and outside Cross River state.

### Sample collection

Fresh periwinkles were purchased from Watt and Marian markets within Calabar Metropolis. Three lines of vendors in the periwinkle section of each market were selected for the study. One cup of periwinkle containing between 75 and 90 individuals (depending on size) was purchased from each of five randomly selected vendors on a periwinkle line (every 5^th^ vendor on a line of between 25 and 29 vendors). The five samples from each line were pooled together and designated sampling point 1, 2 and 3 for line 1, 2 and 3, respectively. The samples were transported in polyethylene bags to the Zoology and Environmental Biology Laboratory, University of Calabar, Calabar, Nigeria. Six samples were therefore obtained each month (one from each of the three sampling points in each of the markets). Sampling was conducted once monthly for three months from June to August, 2019, bringing the total number samples to eighteen (obtained from 90 vendors).

### Sample preparation

The weights of the periwinkles from each sampling point per month were measured. Only fully matured periwinkle with weights ranging between 5.5 g to 7.5 g were used for the sampling station for that month to minimize the variations in metal concentrations in relation to body size. The concentration of metals in bivalve mollusks could vary with body size.^12^ The edible parts of the periwinkle from each sampling point per month were obtained by cracking the shells. The soft tissues (edible part) of the periwinkle were extracted, dried in the oven (heat drying oven, model DHG) at 80°C for 72 hours to a constant weight and ground into powder. A total of 5 g of the ground sample was placed in a beaker and digested with 20 ml of concentrated nitric acid/perchloric acid mixture in the ratio 3:1 on a hot plate. Concentrated nitric acid was added as necessary until digestion was completed, as shown by a clear solution. The digest was then filtered into 50 ml volumetric flask and made up to the mark with distilled deionized water.

### Metal analysis

The metal concentration in the digests was determined by atomic absorption spectrophotometry (Shimadzu, model AAS-6800, Japan) equipped with Zeeman background correction and graphite furnace at the National Research Institute for Chemical Technology, Zaria. The calibration curve was prepared by running different concentrations of the standard solutions.

### Analytical quality assurance

Appropriate quality assurance procedures and precautions were taken to ensure the authenticity of the results. Samples were carefully handled to avoid cross-contamination. Glassware was properly cleaned and distilled deionized water was used throughout the study. Reagents used – nitric acid (Riedel-de Haen, Germany) and perchloric acid (British Drug Houses Chemicals Limited, England) were of analytical grade. In order to check the reliability of the analytical method employed for metal determination, one blank and combined standards were run with every batch of samples to detect background contamination and monitor consistency between batches. The results of the analysis were validated by digesting and analyzing standard reference materials (animal blood coded IAEA-A-13) following the same procedure. The analyzed values and the certified reference values of the elements determined were compared to ascertain the reliability of the analytical method employed.

### Statistical analysis

Test for normality was carried out using the Shapiro–Wilks test and the Z-score test was used to check for outliers. Having passed the test for normality and outliers, data collected were subjected to statistical test of significance. Independent t-test was used to compare metal concentrations in *Tympanotonus fuscatus* between the two markets. Probabilities less than 0.05 (*P* < 0.05) were considered statistically significant. Analysis of variance (ANOVA) test was used to assess significant variation in metal concentration between the sampling months. Probabilities less than 0.05 (*P* < 0.05) were to be considered statistically significant. The Duncan multiple test or Donnette T was adopted for multiple comparison between sampling months depending on whether the homogeneity test was greater or less than 0.05.

### Estimated daily intake

The EDI of metals from edible tissues of *Tympanotonus fuscatus* in this study was determined using the method by Ado *et al*., expressed in Equation 1.^13^

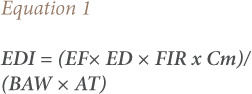
where, EF is the exposure frequency (365 days/year), ED is exposure duration (adopted from Oguguah *et al, 2017*^14^ as 54.5 years equivalent to average life time expectancy for a Nigerian adult), FIR is the fish ingestion rate (the FIR for Nigerians of 0.02 kg/person/day was also adopted from Oguguah *et al*., and used for edible tissues of *Tympanotonus fuscatus*), Cm is the concentration of metal in edible tissues of *Tympanotonus fuscatus* (mg/kg), BAW is the average body weight for an adult (60.7 kg) and AT is the average exposure time-age (EF x ED).^14^

The fish ingestion rate (0.02 kg/person/day) applied to fresh fish, the concentrations of metals measured in this study referring to dry weight were recalculated to fresh weight based on the available information on the mean moisture content of periwinkle from the area, to ensure consistency between the unit used for fish ingestion rate and measured concentration data. This was done following the United States Environmental Protection Agency (USEPA), Office of Research and Development, National Centre for Environmental Assessment's guidance and risk assessments for intake of fish and shell fish.^15^ The conversion of metal concentrations measured in dry weight to wet weight was done using a moisture content percentage of 13.45^8^ according to Equation 2.^15^

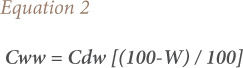
where, *Cww* is the wet weight concentration, *Cdw* is the dry weight concentration and *W* is the moisture content.

### Target hazard quotient

Estimation of potential hazard to human health (THQ) through the consumption of edible tissues of *Tympanotonus fuscatus* was computed using Equation 3.



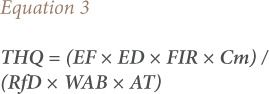
where, R*f*D is the oral reference dose for metal (mg/kg body weight per day) and RfD is an estimate of daily oral exposure for the human population which does not cause harmful or damaging effects during a lifetime.^16^ The methodology for estimation of THQ was adopted from the USEPA Regional Screening Levels (RSLs) – Generic tables, 2020.^17^ The value of RfD for Pb (0.0035 mg/kg per day) was obtained from the literature.^18–21^ The RfD values for Cd (0.001 mg/kg per day), Cr (1.5 mg/kg per day) and Ni (0.02 mg/kg per day) were taken from the USEPA's integrated risk information system.^22^

### Hazard index

The HI was computed as the sum of the THQ of the heavy metals under study as described in Equation 4.^16^

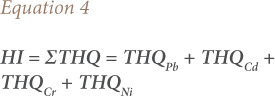



## Results

To evaluate the accuracy and precision of the employed analytical procedure, standard reference materials of animal blood coded IAEA-A-13 were analyzed in a like manner to our samples. The analyzed values and the certified reference values of the elements determined were very close, suggesting the reliability of the method employed *(Table 1).*

**Table 1:** Results of Analysis of Reference Material (Animal Blood IAEA-A-13) Compared to the Certified Reference Value (mg/kg)

**Element (mg/kg)**	**Pb**	**Ni**	**Cr**	**Cu**	**Cd**
Analyzed value	5.25	1.20	4.45	4.00	0.140
Reference value	4.2–5.5	1.00–1.50	4.30–5.00	3.1–4.1	0.1–2.34

### Metal concentrations in edible tissues of *Tympanotonus fuscatus*

Results obtained from the determination of heavy metal contents of edible tissues of *Tympanotonus fuscatus* purchased from the two major markets (Watt Market and Marian Market) in Calabar Metropolis for June, July and August are presented in Table 2.

**Table 2:** Metal Concentration (mg/kg dw) in Edible Tissues of Tympanotonus fuscatus Obtained from Watt Market and Marian Market, Calabar Metropolis, Nigeria

	**SP**	**Watt Market (N = 9) (mg/kg dw)**				**Marian Market (N = 9) (mg/kg dw)**			

		**Pb**	**Cd**	**Cr**	**Ni**	**Pb**	**Cd**	**Cr**	**Ni**
**June**	1	0.037	0.028	0.043	0.085	0.043	0.032	0.035	0.038
	2	0.056	0.018	0.048	0.221	0.032	0.021	0.049	0.321
	3	0.032	0.032	0.157	0.261	0.052	0.031	0.031	0.322
	Mean±SD	0.042±	0.026±	0.083±	0.189±	0.0423±	0.028±	0.038±	0.227±
		0.013^a^	0.01 ^a^	0.06^a^	0.09^a^	0.010^a^	0.006^a^	0.01^a^	0.16^a^
	Range	0.032	0.018	0.043	0.085	0.032	0.021	0.031	0.038
		0.056	0.032	0.157	0.261	0.052	0.032	0.049	0.322
**July**	1	0.031	0.016	0.041	0.211	0.033	0.021	0.038	0.217
	2	0.028	0.016	0.058	0.243	0.029	0.011	0.052	0.211
	3	0.011	0.019	0.039	0.221	0.021	0.017	0.026	0.201
	Mean±SD	0.023±	0.017±	0.046±	0.225±	0.028±	0.016±	0.039±	0.209±
		0.014^a^	0.008^ab^	0.01^a^	0.02^a^	0.006^ab^	0.005^a^	0.01^a^	0.01^a^
	Range	0.011	0.016	0.039	0.211	0.021	0.011	0.026	0.201
		0.031	0.019	0.058	0.243	0.033	0.021	0.052	0.217
**August**	1	0.019	0.008	0.134	0.076	0.024	0.019	0.018	0.026
	2	0.012	0.011	0.021	0.058	0.022	0.018	0.018	0.017
	3	0.034	0.016	0.014	0.053	0.009	0.025	0.012	0.012
	Mean±SD	0.023±	0.012±	0.056±	0.062±	0.018±	0.021±	0.016±	0.018±
		0.011^a^	0.004^a^	0.07^a^	0.01^b^	0.008**^b^**	0.004^a^	0.00^b^	0.01^a^
	Range	0.012	0.008	0.014	0.053	0.009	0.018	0.012	0.012
		0.034	0.016	0.134	0.076	0.024	0.025	0.018	0.026

Abbreviation: SP, sampling point

The lead concentration of edible tissues of *T. fuscatus* from Watt Market ranged from 0.011 to 0.056 mg/kg. The lowest value was observed in July and the highest value in June. Statistical analysis revealed no statistically significant (ANOVA, *P* > 0.05) difference in Pb concentration for *T. fuscatus* from Watt Market across the three months in the present study. On the other hand, Pb concentration of edible tissues of *T. fuscatus* from Marian Market ranged from 0.009 to 0.052 mg/kg. The lowest value was observed in August and the highest value in June. The difference in Pb content of *T. fuscatus* across the three months was statistically significant (ANOVA, *P* < 0.05), with the Pb concentration measured in the month of June significantly higher than August. The difference between June and July, and between July and August was not significant at a 95% confidence level.

The cadmium concentration of edible tissues of *T. fuscatus* from Watt Market ranged from 0.008 to 0.032 mg/kg, with the lowest value observed in August and the highest value in June. Statistical analysis revealed that the difference in Cd concentration of edible tissues of *T. fuscatus* from Watt Market across the three months was statistically significance (ANOVA, P<0.05), with June significantly higher than August. The differences between June and July and between July and August were not significant. Cadmium concentrations in edible tissues of *T. fuscatus* from Marian Market ranged from 0.011 to 0.032 mg/kg. The lowest value was observed in July and the highest in June. The difference in Cd concentration of edible tissues of *T. fuscatus* from Marian Market between the three months was not statistically significant (ANOVA, *P* > 0.05).

The chromium concentration of edible tissues of *T. fuscatus* from Watt Market ranged between 0.014 and 0.157 mg/kg. The lowest value was observed in August and the highest concentration in June. The difference in Cr concentration across the three months was not statistically significant (ANOVA, *P* > 0.05). The chromium content of edible tissues of *T. fuscatus* from Marian Market ranged between 0.012 and 0.052 mg/kg. The lowest value was observed in August and the highest concentration in July. The difference in Cr concentration of *T. fuscatus* from Marian Market across the three months was significant (ANOVA, *P* < 0.05), with the Cr concentration in the month of June significantly higher than August. The chromium concentration in July was also significantly higher than August, but the difference between June and July was not significant.

The nickel concentration of edible tissues of *T. fuscatus* from Watt Market ranged between 0.053 mg/kg and 0.261 mg/kg. The lowest value was observed in August and the highest concentration in June. The difference in Ni concentration of *T. fuscatus* from Watt Market between the three months was significant (ANOVA, *P* < 0.05), with the Ni concentration in June being significantly higher than August.

The nickel concentration in July was also significantly higher than August. The nickel concentration of edible tissues of *Tympanotonus fuscatus* from Marian Market ranged between 0.012 and 0.322 mg/kg. The lowest value was also observed in August and the highest concentration in June. The difference in Ni concentration of *Tympanotonus fuscatus* from Marian Market across the three months studied was not statistically significant (ANOVA, *P* > 0.05).

### Estimated daily intake

In order to assess the health risk of any pollutant, it is necessary to estimate the level of exposure. One very significant aspect of such estimation is the evaluation of daily intake. Average values of EDI (mg/kg body weight (bw)/day) recorded for Watt Market were 0.009, 0.005, 0.018 and 0.045 for Pb, Cd, Cr and Ni, respectively. Average values of EDI (mg/kg bw/day) recorded for Marian Market were 0.008, 0.006, 0.009 and 0.043 for Pb, Cd, Cr and Ni, respectively *(Table 3).*

**Table 3:** Estimated Daily Intake of Metals in Edible Tissues of Tympanotonus fuscatus Purchased from Watt and Marian Markets, Calabar Metropolis

	**Watt Market**				**Marian Market**			
	
	**Lead**	**Cadmium**	**Chromium**	**Nickel**	**Lead**	**Cadmium**	**Chromium**	**Nickel**
June	0.012	0.008	0.024	0.054	0.012	0.008	0.011	0.064
July	0.007	0.005	0.013	0.064	0.008	0.005	0.011	0.060
August	0.007	0.003	0.016	0.018	0.005	0.006	0.005	0.005
*Average*	*0.009*	*0.005*	*0.018*	*0.045*	*0.008*	*0.006*	*0.009*	*0.043*
UL (mg/day)	0.240	0.064	0.130	3–7	0.240	0.064	0.130	3–7
RDI (mg/day)	0.00	0.00	0.003–1.5	0.500	0.00	0.00	0.003–1.5	0.500

Abbreviations: EDI, estimated daily intake; UL, upper tolerable daily intake; RDI, recommended daily intake.

Values expressed as mg/kg bw/day except as otherwise stated.

### Target hazard quotient

The risk to human health by the intake of metal-contaminated *T. fuscatus* was also characterized using the THQ. The THQ is the ratio between exposure and the reference oral dose. Average THQ values for Watt Market were 2.385, 5.272, 0.012 and 2.002 for Pb, Cd, Cr and Ni, respectively. Average THQ values for Marian Market were 2.385, 6.151, 0.006 and 2.147 for Pb, Cd, Cr and Ni, respectively *(Table 4).*

**Table 4:** Target Hazard Quotient of Metals in Edible Tissues of Tympanotonus fuscatus Purchased from Watt Market and Marian Market, Calabar Metropolis

**Months**	**Watt Market**				**Marian Market**			
	
	**Lead**	**Cadmium**	**Chromium**	**Nickel**	**Lead**	**Cadmium**	**Chromium**	**Nickel**
June	3.389	7.578	0.016	2.702	3.389	7.908	0.007	3.196
July	1.883	4.942	0.009	3.213	2.259	4.613	0.007	2.982
August	1.883	3.295	0.011	0.090	1.506	5.931	0.003	0.264
Average	2.385	5.272	0.012	2.002	2.385	6.151	0.006	2.147

### Hazard index

The HI was developed to evaluate the potential risk to human health through more than one heavy metal. The hazard index in the present study was determined to be 9.7 for Watt Market and 10.7 for Marian Market.

## Discussion

There are many exposure pathways of toxic heavy metals to humans and the food chain is considered one of the most important. Globally, the human health risk due to the consumption of food from aquatic ecosystems contaminated with hazardous chemicals including heavy metals has increased.^14^ There is a possibility that the level of these chemicals in seafoods may not have significant adverse effects on an individual organism, but may still have devastating effects on animals that consume them due to bioaccumulation and biomagnification arising from the food chain as a result of the trophic transfer factor.^23^ Fish are the most important single source of high-quality protein contributing over 17% of animal protein and 6.7% of all protein consumed by the world's population. The consumption of fish has increased significantly in recent years due to potential nutritional and therapeutic benefits.^24^ Consequently, humans are potentially exposed to heavy metals and other hazardous chemicals that are non-biodegradable and easily accumulated in fish. In order to effectively assess potential human health risk of Pb, Cd, Cr, and Ni due to the consumption of periwinkle obtained from major markets in Calabar, it is necessary to estimate levels of human exposure by first quantifying the concentration of these metals in edible tissue of the organism and then compare with global regulatory standards.

The mean Pb levels for periwinkle from Watt Market and Marian Market *(Table 2)* were found to be below the FAO/WHO maximum permissible limits of 0.05 mg/kg.^25^ The mean level measured in this study was also below the Joint FAO/WHO Expert Committee on Food Additives recommended maximum levels; the Commission of the European Communities' maximum levels (1.5 mg/kg) for bivalve mollusks; the Codex Committee on Food Additives and Contaminant maximum levels of 0.2 mg/kg.^26,27^ The implication of these findings is that periwinkle obtained from the two major markets in Calabar do not pose significant adverse health impact with respect to Pb poisoning, but there is a serious cause for concern considering the fact that in 2010 the WHO withdrew the provisional tolerable weekly intake for Pb on “the grounds that it is not possible to set an intake value that is protective for health”.^28^ Lead is a persistent metal which has been characterized as a priority hazardous substance. It is a non-essential element, with no known role in living organisms and exhibits extreme toxicity even at very low exposure levels. There is no known safe exposure concentration for lead. The toxicity has greater impact in children than adults because children absorb four to five times as much ingested lead as adults.^29^ Health effects include interference with the development of a child's brain and central nervous system, reduction of IQ, behavioral problems and reduced cognitive development. Lead induced increase in blood pressure, cardiovascular diseases, liver and kidney dysfunction is more common in adults.^30^ A similar mean value (0.02 mg/kg Pb) was reported for periwinkle obtained from the upper reaches of the Bonny Estuary, Nigeria.^31^ In addition, a similar range (0.003–0.032 mg/kg) was recorded for periwinkles obtained from the Great Kwa River, Nigeria.^32^ Lead values ranging from 0.02 to 0.21 mg/kg were recorded for periwinkles obtained from Uta-Ewa creek, Imo River Estuary, Nigeria.^33^ In a study of heavy metals in seafoods and farm produce in Uyo, Nigeria, a higher mean value (0.34 mg/kg) was recorded for Pb in periwinkle.^1^ A higher mean value of 55.88±14.28 was also reported for periwinkle obtained from the Benin River, Nigeria.^34^ Lower values ranging from 0.001–0.004 were recoded for periwinkle harvested from a tropical mangrove forest in the Niger Delta, Nigeria.^35^ The significant difference in Pb concentration observed between the months of June and August could be attributed to dilution. August was the peak of the wet season in 2019. An increase in water levels due to continuous rains may have resulted in the dilution of contaminant levels in the Niger Delta, suggesting that periwinkle could be a bioindicator of this metal.

The presence of Cd in seafood results from the contamination of water. It is known to accumulate in aquatic organisms, particularly detritus feeders such as mollusks.^25^ Cadmium is also not an essential element and the WHO/FAO have determined the maximum tolerable daily intake to be 2 mg/person per day.^36^ The European Commission maximum levels for Cd in bivalve mollusks is 1.0 mg/kg wet weight.^37^ In the current study, Cd was below the permissible levels. At its sixteenth meeting, the WHO committee on food additives and contaminant levels in food allocated a provisional tolerable weekly intake of 0.4–0.5 mg of Cd per person and at its thirty-third meeting this value was expressed in terms of intake per kilogram body weight (0.007 mg/kg bw).^38^ For an average adult body weight (60.7 kg), the provisional tolerable weekly intake is 0.42 mg, which is equal to 0.06 mg per day. Considering the accumulative property and long biological half-life of Cd, the concentration measured in this study is a serious cause for concern. The toxic effects of Cd in food are largely related to long-term exposure to low doses.^38^ Low dietary concentrations of Cd, Zn and Fe have been shown to promote absorption of Cd.^38^ Cadmium has been reported to cause liver and kidney dysfunction as well as softening of bones following long-term exposure.^14^ Cadmium, Cr and Ni belong to Group 1 of the International Agency for Research on Cancer (IARC) classification system with sufficient evidence for carcinogenicity in humans.^39^ The range of Cd concentrations recorded in this study was lower than the range of 0.59–0.93 mg/kg reported for periwinkles obtained from Uta-Ewa creek, Imo River Estuary, Nigeria.^33^ A mean value of 0.02 mg/kg was reported for the upper reaches of the Bonny Estuary, Nigeria.^31^ Lower values (0.001–0.003 mg/kg) were reported for periwinkle harvested from perturbed tropical mangrove forests in the Niger Delta, Nigeria.^35^ Lower values ranging from 0.002–0.015 mg/kg were also reported for periwinkle obtained from the Great Kwa River, Nigeria.^32^ A higher mean value (1.00±0.6 mg/kg) was recorded for periwinkle obtained from the Benin River, Nigeria and for Uyo, Nigeria (0.34 mg/kg).^1,34^ The significant difference in Cd concentration observed between June and August could also be attributed to dilution due to high water volume at the peak of the wet season.

Chromium is an essential element that potentiates insulin action and enhances carbohydrate, lipid and protein metabolism at a maximum intake level of 0.25 mg/day, equivalent to 0.0041 mg/kg body weight per day for an average adult of 60.7 kg.^40, 41^ However, exceeding this limit leads to bioaccumulation and toxicity that can result in hepatitis and ulcers.^1^ It is also assumed to cause cancer.^42^ The findings of this study indicate that Cr concentration measured in periwinkle was lower than the WHO and European Food Safety Authority (EFSA) safe limits. The values recorded were lower than the mean value (1.07 mg/kg) noted in a similar study and 1.57 mg/kg reported for periwinkle obtained from the upper reaches of the Bonny River, but higher than the 0.001–0.003 mg/kg found for periwinkle harvested from a perturbed tropical mangrove forest in the Niger Delta.^1,31,35^ The concentration of Cr recorded in this study was also higher than the range of 0.013–0.157 mg/kg reported for soft tissues of periwinkle obtained from the Great Kwa River, Nigeria.^32^

Nickel is an essential element in animals, although its functional importance in humans has not been clearly demonstrated. It is present in the pancreas and hence plays an important role in the production of insulin. A tolerable daily intake of 2.8 μg Ni/kg bw/day was derived by the EFSA.^43^ For an average adult of 60.7 kg, the provisional tolerable daily intake is 0.012 mg. The nickel level recorded in this study was below the allowable limit. The kidney is the primary target organ for oral exposure of Ni. Other target organs include the cardiovascular system, immune system and blood. Nickel is reported to correlate with increased incidences of cancer in humans.^39^ Values ranging from 0.09–1.07 μg/g were reported for Ni in periwinkle obtained within Uyo metropolis, Nigeria.^1^ These values agree with the findings of the present study except for one sample that recorded a value of 1.07 μg/g. A higher mean value of 8.49±2.54 was reported for Ni in periwinkle obtained from the Benin River, southern Nigeria.^34^ The concentration of Ni recorded in the present study was also higher than the range of 0.000–0.012 mg/kg reported for soft tissues of periwinkle obtained from the Great Kwa River, Nigeria.^32^

### Estimated daily intake

In assessing potential human health risk caused by any chemical contaminant over a prolonged exposure, another very important aspect of estimating level of exposure is by evaluation of daily intake. The EDI, which combines data on contaminant concentrations in foodstuffs and quantity of food consumed on a daily basis is widely used to describe safe levels of contaminants consumed through food.^16,43^ In the present study, approximate Pb, Cd, Cr and Ni intakes for people living in Calabar metropolis through the consumption of periwinkle purchased from two major markets were estimated and compared with the recommended daily intake and the upper tolerable daily intake for the metals *(Table 3).* The tolerable daily intake is an estimate of the amount of a chemical contaminant from all available sources that can be taken in daily over a lifetime without appreciable health risk.^16^ The average EDI of Pb and Cd for periwinkles obtained from both Watt and Marian markets were slightly above the recommended daily intake for the metals, while Cr and Ni were below this limit. The average EDI of the four metals under study (Pb, Cd, Cr and Ni) for periwinkles obtained from both markets were below the upper tolerable daily intake for the metals *(Table 3).* The estimated daily metal intake computed in this study were expressed per kilogram body weight per day (mg/kg bw/day) so that for an average adult of 60.7 kg bw, the average EDI of, for instance Pb in periwinkle from Watt and Marian markets (equivalent to 0.009 and 0.008, respectively) multiplied by 60.7 is equal to 0.546 and 0.486 mg per day, respectively. The results obtained from the EDI of Pb, Cd, Cr and Ni in this study suggest that perennial intake of periwinkle purchased from Watt and Marian markets in Calabar is likely to induce health risks with respect to Pb and Cd intoxication.

### Target hazard quotient

The potential risk to human health by the intake of Pb, Cd, Cr and Ni due to the consumption of periwinkle from major markets in Calabar was also characterized using the target hazard quotient. The THQ is a dimensionless quantity and is defined by the USEPA as the ratio between exposure and reference oral dose.^17,44^ When the ratio is lower than one (1), there is no obvious risk. The THQ method employed in the present study considered only exposure to the metals under study through the consumption of periwinkle purchased from Watt and Marian markets, Calabar without considering other exposure routes. The average THQs of all metals studied were found to be above 1, with Cr being the only exception. This implies that consumption of periwinkle purchased from the two markets could pose toxicological risk with respect to Pb, Cd and Ni poisoning.

### Hazard index

The hazard index expressed as the sum of the target hazard quotient of the metals under study was used to evaluated potential health risk due to all the metals combined.^16,22^ Hazard index assumes that the severity of adverse effect of metals poisoning is proportional to the sum of the multiple metals exposure. Significant potential health risk is implied when the HI is greater than 1.^16^ Even though there was no apparent risk when each metal was analyzed individually, the potential risk could be multiplied when all metals are considered together. The HI for a typical adult of 60.7 kg bw considered in this study was found to be 9.7 for Watt market and the relative contributions to the aggregated risk were 24.66%, 54.51%, 0.0001% and 20.70% for Pb, Cd, Cr and Ni, respectively. The hazard index for Marian market was 10.7. The relative contributions to the aggregated risk were 22.31%, 57.55%, 0.06 and 20.09% for Pb, Cd, Cr and Ni, respectively.

## Conclusions

The high protein, omega-3 fatty acids, vitamins, essential minerals and low saturated fat contents of fish and fishery products are known to contribute to good health. However, consumption of fishery products with elevated metal levels poses serious toxicological risk. The findings of this study indicate Pb, Cd, Cr and Ni content of edible tissues of periwinkle obtained from Watt and Marian markets in Calabar metropolis were below FAO, FAO/WHO and Commission of European Communities maximum permissible limits. The average EDI of Pb and Cd were slightly higher compared to the recommended daily intake for the metals. The EDI of the four metals under study (Pb, Cd, Cr and Ni) were lower than the upper tolerable daily intake. The THQ computed to estimate human health risk posed by each metal were above the safe limits of unity, except for Cr. The HI for a typical adult of 60.7 kg bw considered in this study was found to be 9.7 for Watt Market and the relative contributions to the aggregated risk were 24.66%, 54.51%, 0.0001% and 20.70% for Pb, Cd, Cr and Ni, respectively. The HI for Marian Market was 10.7 and the relative contributions to the aggregated risk were 22.31%, 57.55%, 0.06% and 20.09% Pb, Cd, Cr and Ni, respectively.

The present study suggests that consumption of periwinkle from the major markets in Calabar, Nigeria poses toxicological risk with respect to Pb, Cd and Ni poisoning.
